# Neoadjuvant Chemotherapy for Oropharyngeal Cancer Treatment De-Escalation: From Historical Failures to Contemporary HPV-Driven Paradigms

**DOI:** 10.3390/cancers18010023

**Published:** 2025-12-21

**Authors:** Alvaro Sanabria, Juan P. Rodrigo, Anna Luíza Damaceno Araújo, Luiz P. Kowalski

**Affiliations:** 1 Department of Surgery, School of Medicine, Universidad de Antioquia-Hospital Universitario San Vicente Fundacion-Clinica Somer-Clinica Las Vegas Grupo Quironsalud, Medellín 050010, Colombia; 2 Department of Otolaryngology, ISPA (Instituto de Investigacion Santiara del Principado de Asturias), IUOPA (Instituto Universitario de Oncología del Principado de Asturias), CIBERONC (Centro de Investigación Biomédica en Red de Cáncer), Hospital Universitario Central de Asturias, University of Oviedo, 33011 Oviedo, Spain; jprodrigo@uniovi.es; 3 Head and Neck Surgery Department, University of São Paulo Medical School, São Paulo 05403-000, São Paulo, Brazil; anna.damaceno@einstein.br (A.L.D.A.); lp_kowalski@uol.com.br (L.P.K.); 4 Head and Neck Surgery Department, Hospital Israelita Albert Einstein, São Paulo 05652-900, São Paulo, Brazil; 5 Department of Head and Neck Surgery and Otorhinolaryngology, A.C. Camargo Cancer Center, São Paulo 01532-001, São Paulo, Brazil

**Keywords:** oropharyngeal squamous cell carcinoma, neoadjuvant chemotherapy, HPV-positive OPSCC, transoral surgery, de-escalation, organ preservation, immunotherapy

## Abstract

**Background/Objectives**: Oropharyngeal squamous cell carcinoma (OPSCC) management has shifted following recognition of HPV-driven disease. Neoadjuvant chemotherapy (NAC) has historically failed to improve overall survival (OS) in mixed head and neck cohorts, although contemporary HPV-stratified series suggest NAC may enable treatment de-escalation. We aimed to narratively synthesize OPSCC-specific evidence on NAC focusing on primary and nodal response, pathologic complete response (pCR), survival, and functional outcomes. **Methods**: We conducted a narrative review of PubMed, selecting primary studies in which OPSCC outcomes were reported separately (surgery- or chemoradiotherapy [CRT]-based strategies; HPV status when available). We extracted study design, treatment regimens, response outcomes, survival, and toxicity data. **Results**: Pre-HPV studies showed variable responses and no consistent OS advantage over locoregional therapy. In the HPV era, non-comparative cohorts of NAC followed by transoral surgery reported substantial downstaging and high pCR rates at both the primary site and regional nodes, with 3–5-year OS frequently ≥80%. NAC+CRT paradigms demonstrated high clinical CR rates and OS exceeding 80–90%, and lower feeding-tube dependence and reduced swallowing morbidity in de-escalated regimens. Comparative retrospective series suggest NAC + surgery may be associated with lower rates of distant metastases and feeding-tube use compared with CRT or upfront surgery, although interpretation is limited by selection bias, regimen heterogeneity, and small sample sizes. **Conclusions**: While randomized trials have not established an OS advantage for NAC over standard CRT in head and neck cancer overall, HPV-positive OPSCC shows emerging evidence that systemic intensification with NAC may enable surgical and/or radiation de-escalation with promising oncologic and functional outcomes.

## Simple Summary

Oropharyngeal cancer is becoming more common in individuals with fewer comorbidities. Traditional therapies, which include surgery, radiation, and chemotherapy, can control cancer but may have serious side effects, such as problems with swallowing. This study examines existing data on using chemotherapy before the primary treatment (neoadjuvant chemotherapy) to reduce tumors and make subsequent treatments less aggressive. By synthesizing the findings from several trials, we investigate whether this approach could keep optimal survival while enhancing quality of life. The findings suggest that neoadjuvant chemotherapy could help in the selection of individuals for a more conservative surgery or radiation, thus decreasing complications and maintaining function. Further clinical trials are needed to validate these advantages and determine which patients will benefit the most.

## 1. Introduction

Oropharyngeal squamous cell carcinoma (OPSCC) is a major public health problem, with incidence rising among young people. The therapeutic landscape for this condition has changed dramatically, owing to the recognition of human papillomavirus (HPV) as a key etiological factor. Traditional treatments, which include surgery (S), radiotherapy (RT), and chemotherapy (CT), have been associated with significant functional and cosmetic morbidity. However, the markedly improved survival of patients with HPV-positive OPSCC has shifted interest toward treatment strategies that preserve oncologic outcomes while optimizing quality of life [1].

Transoral surgical techniques (transoral surgery (TOS), transoral robotic surgery (TORS), or transoral endoscopic ultrasonic surgery (TOUSS)) [2,3] have gained prominence as a way to improve functional outcomes while reducing the morbidity associated with traditional open surgery [4]. These approaches have demonstrated effective oncologic control with better preserving function and quality of life (QOL) in patients with primary OPSCC [5]. However, many patients with advanced tumors are poor candidates for upfront surgery. Therefore, there is increasing emphasis on neoadjuvant approaches. Neoadjuvant chemotherapy (NAC), which includes administering systemic therapy prior to definitive local therapy, may improve outcomes by controlling micrometastatic disease, reducing primary tumor volume, limiting surgical extent, and facilitating adjuvant treatment de-escalation (educing surgical or radiotherapy intensity while maintaining oncologic control) [6,7,8,9].

NAC in head and neck squamous cell carcinoma (HNSCC) has been repeatedly questioned in the literature. Haddad et al. [10] in the PARADIGM study and Cohen et al. [11] in the DECIDE trial evaluated the clinical efficacy of NAC in patients with HNSCC and found no significant improvement in overall survival (OS). However, 55% patients with OPSCC were enrolled in the PARADIGM trial without accounting for HPV status, and only 41 patients with OPSCC were analyzed in the DECIDE study’s subgroup analysis by subsite and HPV association. In both cases, studies were underpowered to detect differences in OS. Other randomized controlled trials (RCTs) and meta-analyses have not demonstrated a consistent OS advantage over upfront locoregional treatment, and concomitant chemoradiotherapy (CRT) remains the regimen with a confirmed benefit [12,13,14]. Taxane-based regimens may yield higher response rates than platinum-only regimens, but are associated with increased hematologic toxicity [13,15]. Furthermore, "chemoselection" based on induction response shows high sensitivity but low specificity, which limits its predictive value for CRT selection [16].

A key counterargument is that many NAC studies pooled heterogeneous anatomic subsites (oral cavity, oropharynx, hypopharynx, and larynx) and were performed before HPV stratification, which may obscure subsite or biology-specific effects [17]. Landmark data indicate that HPV status strongly influences prognosis in OPSCC, suggesting that mixed-subsite trials understated effects within defined subgroups [13]. Moreover, NAC enabled organ preservation in laryngeal/hypopharyngeal tumors without compromising survival [18], a clinically meaningful benefit not captured by OS alone, and it may reduce the risk of distant metastases (DM), suggesting a potential role in patients at high risk of systemic recurrence [19]. Furthermore, NAC may function as a biologic “selection test”, as induction response provides independent prognostic information that could guide definitive therapy [20].

Recent studies have evaluated NAC in OPSCC and have reported encouraging organ-preservation outcomes [21,22,23,24,25]. Furthermore, the introduction of immunotherapy has created new opportunities, with ongoing clinical trials looking at the use of immune checkpoint inhibitors in this scenario [26,27]. However, whether NAC confers differential therapeutic benefit in HPV-positive versus HPV-negative OPSCC remains uncertain [28].

The objective of this review is to provide a historical perspective and synthesize current evidence on the usefulness of NAC in the treatment of OPSCC, with special emphasis on the response rate of the primary tumor and neck metastases. We structured our analysis around a central question: to what extent NAC may allow safe de-escalation of surgical or radiation therapy in OPSCC without compromising oncologic or functional outcomes.

## 2. Materials and Methods

This is a narrative synthesis. A PubMed search was conducted using the terms “oropharyngeal squamous cell carcinoma”, “neoadjuvant chemotherapy”, “induction chemotherapy”, “human papilloma virus”, and “transoral surgery”. Studies were included if they reported outcomes of NAC in OPSCC and provided OPSCC-specific data within surgical or CRT-based treatment strategies. We excluded studies in which OPSCC data could not be disaggregated from other head and neck subsites, as well as reviews without original data and individual case reports. Although a formal risk-of-bias assessment was not made due to the narrative design, we evaluated each study for sample size, HPV stratification, and clarity of outcome reporting.

## 3. Literature Review

The role of NAC in advanced HNSCC has been investigated for decades and remains a subject of debate [29]. Historically, primary treatment modalities for OPSCC included surgery with or without adjuvant RT or CT.

### 3.1. The First Attempts

In the early 1980s, NAC began to be used in advanced OPSCC with the aim of reducing DM, improving local control, and avoiding “commando” surgeries. At that time, the causal and prognostic role of HPV in OPSCC had not yet been recognized.

Deitmer et al. [30] evaluated NAC using cisplatin, bleomycin, and methotrexate followed by leucovorine rescue in 63 patients with SCC (27 oropharyngeal); 77% achieved ≥50% tumor reduction. Calais et al. [31] examined NAC in 135 oral cavity and OPSCC tumors. A total of 34% of tumors were T3–T4. The objective response rate was 45%, including 6% of CR. Cisplatin-based NAC before locoregional treatment was evaluated in 138 patients with T3–T4 OPSCC by the same group [32]: 52 patients received RT alone and 86 received NAC+RT. A total of 34% of patients responded objectively to NAC, and both groups had similar 5-year OS. After receiving NAC (platinum-based), Rohrmeier et al. [33] reported a 10% CR and 12% PR in 41 patients with inoperable T3-T4 SCC of the oropharynx. Bettinger et al. [34] evaluated the histopathologic response to NAC with bleomycin and cisplatin in 43 advanced oral and OPSCC patients. Tumor reduction >50% occurred in 81% of patients, although no patient achieved a pCR. Pfister et al. [6] examined how NAC (cisplatin+ vinblastine, cisplatin+ bleomycin or cisplatin+ 5-FU) and radiation preserved organ function in advanced OPSCC patients. Laryngectomy was avoided in 94% of patients and tongue surgery in 73%. The OS rate at 2 years was 58%. Nathu et al. [7] examined NAC+RT for T4 OPSCC. A total of 26 patients received RT after NAC (cisplatin and 5-FU). The CR of the primary tumor and neck lymph nodes was 35% and 50%, respectively.

All these studies (Table 1) were retrospective, did not include concurrent comparator groups, and did not stratify outcomes by HPV status. NAC showed varying effectiveness in terms of tumor reduction and OS rates (CR and PR rates were around 34% and 80%, respectively). However, no consistent OS benefit was found when compared to RT alone. Despite clinical response, histologic evaluation frequently revealed residual tumors, especially in bigger or endophytic tumors. Functional preservation was a considerable advantage in certain patients, particularly to prevent extensive procedures.

Domenge et al. [35] did the first multicenter RCT comparing NAC (cisplatin and 5-FU) followed by locoregional treatment to locoregional treatment (either of surgery+RT or exclusive RT) in OPSCC patients. The primary outcome was OS, with secondary endpoints including event-free survival, locoregional control and DM. This multicenter trial enrolled 318 patients diagnosed between 1986 and 1993, 83% of whom were in the tonsil of the base of the tongue (BOT) and 74% of whom were classified as stage III or IV. They found a statistically significant difference in OS in the NAC group (absolute OS benefit at 5 years = 12%; median OS 5.1 years in the CT group versus 3.3 years in the control group), but not in event-free survival. Stratification of the results on the type of local treatment, surgery+RT or RT alone, did not reveal any heterogeneity in the effect of NAC. A total of 56% of patients had an objective response (20% CR and 36% PR > 50%). The trial was terminated early due to poor accrual after six years. The results were ultimately published in December of 2000. The authors determined that *"these results are not convincing enough to establish neoadjuvant chemotherapy with cisplatin and fluorouracil as a standard treatment”*. 

At the same time, a group from the same center designed and developed the MACH-NC meta-analysis, whose results were published in March 2000, showing that NAC offered no advantage in terms of OS for HNSCC, which may have contributed to decreased enthusiasm for NAC and early termination of studies [29]. In retrospect, it is reasonable to claim that the MACH-NC meta-analysis had an unfair influence on the NAC approach in OPSCC. First, the meta-analysis included advanced HNSCC from various subsites (larynx, oral cavity, oropharynx, and hypopharynx), which were clinically heterogeneous; second, the differential prognosis between HPV-positive and HPV-negative OPSCC was unknown at the time. A closer examination of subsite-specific data of the original publication) shows that NAC had a small advantage in OS for OPSCC. Two bigger subsequent meta-analyses [17,36] support the earlier results reported by Pignon et al. [29]. However, these analyses pooled all subsites and did not stratify by HPV status.

### 3.2. Studies Published After Domenge, RCT and MACH-NC Meta-Analysis Without Data About HPV

Following the trial by Domenge et al. [35] and the MACH-NC meta-analysis [29], several additional studies reported outcomes of NAC in OPSCC. Most were retrospective case series.

Mantz et al. administered platinum-based NAC followed by concurrent CRT to 61 patients with locoregionally advanced OPSCC [8]. CR to NAC occurred in 65% of patients. Only five patients ultimately required radical surgery for disease control. Machtay et al. [9] examined 53 stage III or IV resectable OPSCC patients. After NAC (carboplatin and paclitaxel), nonresponders proceeded to surgery followed by RT, whereas responders received cisplatin-based CRT. NAC resulted in a 89% response rate, including 13% CR at the primary site. Organ preservation was achieved in 77% of patients. Finnegan et al. evaluated NAC (cisplatin+ 5-FU) followed by hyperfractionated RT with concurrent CT in patients with T3–T4 OPSCC [37]. A total of 17 patients (74%) experienced >50% tumor reduction. Two- and five-year OS rates were 71% and 55%, respectively. Prestwich et al. [38] evaluated 41 patients treated with NAC (cisplatin +5-FU) followed by either cisplatin-based CRT (73%) or RT alone (27%). CR and PR disease rates were 10% and 68%, respectively. The 3-year local and regional control rate for complete responders was 91%. The 3-year DFS and OS were 75% and 66%. Urba et al. [39] investigated organ-preservation strategies for stage II–IV resectable BOT cancers. In the trial, 37 patients underwent two cycles of NAC (cisplatin and 5-FU) followed by concurrent CRT if their primary tumor reduced more than 50%. A ≥50% reduction in tumor size occurred in 81%, and 78% avoided surgery.

Across studies, NAC had high partial and complete clinical response, reduced tumor size, and facilitated organ preservation (Table 2). Three- to five-year OS ranged from 55% to 70%. Even with these encouraging outcomes, CT regimens, patient variables, and tumor sites influenced the results. Mucositis and dysphagia remained common toxicities. None of these studies adjusted outcomes for HPV status.

### 3.3. Non-Comparative Studies Published After the Recognition of HPV as a Prognostic Factor

#### 3.3.1. NAC + Surgery

After a long pause, in 2016, Sadeghi et al. [21] evaluated NAC followed by TORS as a definitive treatment for OPSCC. This case series included 17 patients with resectable OPSCC. After three rounds of NAC (docetaxel, cisplatin, or carboplatin), patients received TORS. Nine patients had tumors on the BOT, four in the tonsils, ten were HPV+ and eight and eleven were classified as T3-4 and N2, respectively. NAC resulted in a mean reduction of 82% in primary tumor volume. pCR occurred in 54% at the primary site and a pCR in regional lymph nodes was observed in 57%. Three-year OS was 94.1%. Park et al. [22] and Solimeno et al. [40] examined the effectiveness of NAC (Cisplatin + TS-1 (Tegafur/Gimeracil/Oteracil), every 21 days) with TORS and tailored adjuvant treatment for locally advanced (T3-T4) OPSCC. The study included 31 patients. Tumors were mostly in the tonsils (27 patients) and BOT (4 cases). Most patients (51.6%) had T3 tumors, whereas 67% were HPV+. After NAC, 9.7% patients had aCR and 90.3%, a PR. The study did not report specific pCR rates following TORS. Overall, 5-year OS was 78.7% and DFS was 80.8%. Most patients (87%) subsequently received adjuvant RT. The 5-year OS rate for p16+ and p16- OPSCC was 90% and 70%, respectively. In a larger follow-up series of 308 patients (including 197 with OPSCC, 67% HPV-positive), Costantino et al. [41] reported rates pCR of 32.8% at the primary tumor and 43% in regional lymph nodes. The 3-year OS and DFS rates were 88.7% and 81,4%, respectively. Kim et al. [42] evaluated the correlation between radiological and pathological responses to NAC (cisplatin + 5-FU) followed by TORS in HPV-positive carcinoma. The research included 38 patients with tonsillar tumors who underwent surgery. A total of 18.4% patients were classified as T3/T4, and 26.1% patients were classified as N2-3. pCR occurred in 60% of primary tumors and 45.5% of regional lymph nodes. Patients achieving pCR after NAC required significantly less adjuvant therapy. The 5-year OS for the cohort was 81.1%. Sadeghi et al. [43] conducted a prospective trial evaluating pCR to NAC (cisplatin + docetaxel) followed by surgery in patients with HPV-positive OPSCC. The study included 54 patients with tumors at the BOT and tonsils. Most patients had T1-2 disease (69%) and N2 nodal involvement (72%). Of the patients, 72% had a pCR at the primary site and 57% in the neck (Table 3).

Across studies, NAC demonstrated encouraging oncologic and functional outcomes. In HPV-positive tumors, pCR rates ranged between 20% and 72% at the primary site. The 3- to 5-year OS ranged between 58 and 94%, while DFS exceeded 80%. Functional recovery was generally rapid and required little long-term tracheostomy or feeding-tube support. However, these findings must be interpreted cautiously. Most studies are single-center case series or early-phase prospective trials with small sample size and substantial heterogeneity in patient selection (HPV status, T stage), CT regimens, and surgical approaches (TORS, TLM, open surgery). Follow-up durations remain insufficient to fully assess late recurrences or long-term functional sequelae.

#### 3.3.2. NAC + CRT

Marur et al. [44] conducted a phase II trial (E1308) to assess the efficacy of NAC combined with reduced-dose radiation and weekly cetuximab for patients with stage III-IVB HPV-positive OPSCC. The study included 80 patients. Patients received three cycles of NAC (cisplatin, paclitaxel, and cetuximab). Patients who achieved cCR at the primary site received reduced-dose IMRT (54 Gy) with weekly cetuximab. Those without cCR at the primary site or lymph nodes received standard-dose radiation (69.3 Gy) to those regions. Of the 80 eligible patients, 77 finished all three cycles of NAC. Among them, 70% achieved a cCR at the primary site and 58% at nodal site. Among all evaluable patients, 2-year PFS and OS were 78% and 91%, respectively. In the subgroup treated with 54 Gy after cCR, 2-year PFS and OS were 80% and 94%, respectively. Dose-reduced radiation was associated with improved swallowing function and better nutritional outcomes. Misiukiewicz et al. [45] reported the Quarterback trial, a phase II/III trial comparing standard-dose CRT (sdCRT) with reduced-dose CRT (rdCRT) following NAC in 20 patients with stage III-IVB HPV-positive OPSCC. All patients received NAC with docetaxel, cisplatin, and 5-FU. After NAC, 80% patients had CR in the primary tumor. In the neck lymph nodes, 80% patients had a CR. Three-year OS was 87.5% vs. 83.3% for sdCRT and rdCRT, respectively. Seiwert et al. [46] studied the effectiveness and safety of dose and volume de-escalation in HPV-positive OPSCC (phase II study OPTIMA). The study included 62 patients with stage III-IVB HPV-positive OPSCC. The trial implemented a three-tiered, response-adaptive de-escalation strategy using carboplatin and nab-paclitaxel. Low-risk patients (≤T3, ≤N2b, ≤10 pack-years) achieving ≥50% response received reduced-dose RT (50 Gy; RT50) while low-risk patients with 30%-50% response or high-risk patients (T4 or ≥N2C or >10 pack-year) with ≥50% response received 45 Gy+ paclitaxel-based CRT (CRT45). Patients with lesser response received standard-of-care 75 Gy+ paclitaxel-based CRT (CRT75). Most patients responded to NAC (71% with reduction > 50% and 21% between 30 and 50%), similar between risk groups. Neck surgical specimens demonstrated pCR in 90% of patients. The 2-year OS was 98%. The 2-year PFS and OS were 95% and 100% for low-risk patients and 94% and 97% for high-risk patients, respectively. Rosenberg et al. [47] updated the cohort to 90 patients, confirming similar outcomes. In a phase 2 non-randomized clinical study (OPTIMA II), Rosenberg et al. [26] included 73 patients with HPV-positive OPSCC who received neoadjuvant nivolumab combined with CT (nab-paclitaxel and carboplatin) followed by response-adaptive treatment. The tumors were in stages III-IV. Subsequent therapy was assigned using a risk- and response-adaptive framework: group A, single-modality RT alone or TORS (28 patients); group B, intermediate dose CRT of 45 to 50 Gy (34 patients); and group C, regular dose CRT of 70 to 75 Gy (10 patients). Among 73 evaluable patients, 70.8% achieved ≥50% tumor reduction. Two-year OS was 91.4%. By response-adapted group, 2-year PFS and OS for group A were 96.4% and 96.4%, and group B, 88.0% and 91.0%, respectively. Lower dependence on enteral feeding and improved swallowing outcomes were observed among patients who received response-adapted therapy (Table 4).

Across these trials, response rates to NAC in advanced HPV-positive OPSCC varied, with PR rates of 70–88% and CR rates of 13–67%, depending on CT regimen and patient selection. The studies reported 2-year progression-free survival and OS rates of more than 80%. Incorporation of immunotherapy appears to enhance response rates while preserving functional outcomes.

### 3.4. Comparison Between NAC + Surgery vs. CRT

Comparative, non-randomized studies have explored whether NAC followed by surgery offers advantages over definitive CRT in HPV-positive OPSCC.

Sadeghi et al. [23] evaluated the efficacy of NAC (cisplatin + docetaxel) followed by surgery compared with CRT in HPV-positive OPSCC using a matched cohort (NECTORS). The analysis included 55 patients in the NAC + surgery group and 142 in the CRT group. A total of 30% had T3-T4 disease and 80% had N2-3 disease, with tumors located in the BOT and tonsils. cPR occurred in 43.6% of patients in the NAC + surgery group. Five-year DFS was 96.1% in the NAC + surgery cohort compared to 69.9% in the CRT cohort. Feeding-tube dependence was markedly lower in the NAC + surgery group (0% vs. 24%). DM was less frequent in the NAC + surgery group (0% vs. 12%). An updated analysis in 2024 including 209 patients reported similar findings [48].

These trials suggest that systemic intensification with NAC, combined with surgical de-escalation, may reduce DM and potentially improve survival and functional outcomes. However, important limitations constrain interpretation. The NAC+S arms had relatively small sample sizes (55 and 110 patients) compared with historical CRT controls, introducing the risk of selection bias. Follow-up duration was shorter in the NAC + surgery cohorts. Furthermore, the non-randomized design precludes definitive conclusions regarding OS benefit.

### 3.5. Comparison Between NAC + Surgery vs. Upfront Surgery + CRT

Bang et al. [24] compared oncologic and functional outcomes of NAC followed by surgery with those of primary surgery in patients with HPV-positive tonsillar carcinoma. The study included 112 patients, with the majority classified as T1-2/N+. Patients were separated into two groups: The NAC + Surgery group (n = 38) received two cycles of NAC (docetaxel, cisplatin, and 5-FU), followed by surgery and adjuvant treatment based on pathological findings, as well as upfront surgery (n = 74) that included primary surgical resection of the tumor and adjuvant treatment based on the final pathology report. However, N stage distribution differed between groups, with the NAC cohort having a higher proportion of N2–N3 disease. Among 38 patients receiving NAC, cCR occurred in 61% at the primary site and 45% in regional lymph nodes. NAC was associated with substantial downstaging in both T and N categories and with fewer close or positive surgical margins. The 5-year OS did not differ significantly between the two groups (72.9% for NAC + S vs. 71.0% for upfront surgery); however, DFS improved in the NAC + S group (86% versus 63.7%). Patients in the NAC + S group resumed oral intake sooner and were more often able to maintain a regular or soft diet. They also underwent fewer free-flap reconstructions and required less adjuvant RT. Sampieri et al. [25] compared NAC (two cycles of cisplatin) and TS-1 (tegafur, Gimeracil, and oteracil) followed by TORS to upfront TORS for stage III-IV OPSCC in 204 patients after matching (102 in the NAC + TORS group and 102 in the upfront TORS group). The tonsils were the most common tumor location (77%), followed by the BOT (22%). Most patients (74%) were HPV-positive, 16% were T3-4 and 67% were N2. pCR occurred in 27% of the NAC group and 33% of the upfront-surgery group. In the HPV-positive subgroup, NAC + TORS was associated with markedly higher downstaging rates (93% vs. 45%), fewer positive margins (11% vs. 42%) and a markedly lower need for adjuvant therapy (16% vs. 51%). In the HPV-negative subgroup, NAC showed numerically favorable trends, although these differences did not reach statistical significance. There were no significant differences between the two groups in terms of recurrence rates or 5-year OS outcomes (OS 91% for NAC versus 88% for the control group).

As discussed below, interpretation is limited by retrospective design and selection bias. Because the CRT cohorts served as historical controls, temporal changes in treatment practices and RT techniques may have introduced confounding. Small sample sizes in the NAC + surgery cohorts further limited statistical power.

## 4. Conclusions

NAC has not consistently demonstrated superiority over upfront locoregional treatment in terms of OS [29,36]. Licitra and Vermoken [49] underlined that many trials reporting no benefit pooled multiple subsites with differing prognoses, potentially diluting site-specific effects. Subsequent syntheses suggest that, outside of larynx-preservation settings [18], evidence that NAC enhances outcomes compared with CRT alone remains inconsistent [50]. Recent HPV-positive OPSCC cohorts [17,21,22,23,29,36,40,41,42,48] suggest that NAC can achieve high pCR rates, facilitate downstaging, and reduce the need for adjuvant RT/CRT, potentially allowing less extensive surgery, lower feeding-tube dependence and better functional recovery. However, HPV-negative OPSCC remains under-investigated, with available evidence largely restricted to heterogeneous, retrospective cohorts.

**Figure 1:**
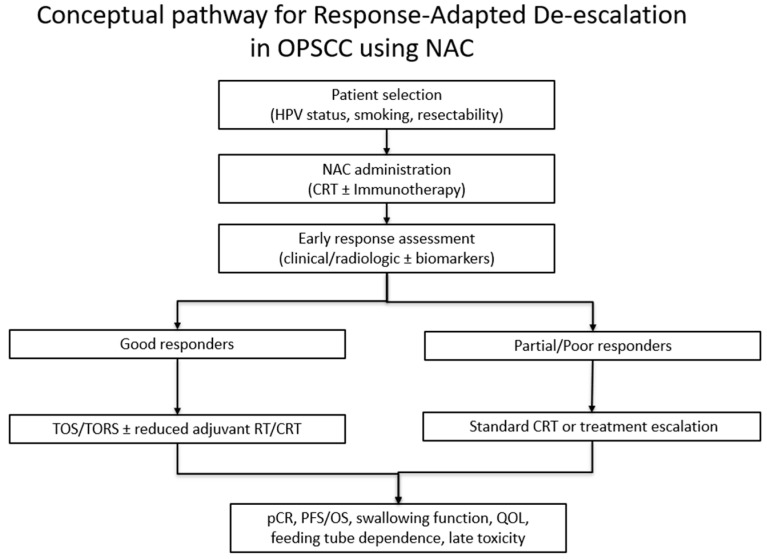
Conceptual pathway for response-adapted de-escalation in OPSCC using NAC.

Interpretation of the current evidence is limited by the predominance of retrospective non-randomized designs, heterogeneity in treatment regimens and inconsistent reporting of HPV status. Functional outcomes such as feeding-tube dependence and QOL are reported with substantial variability in timing, measurement, and use of validated instruments, which limits the ability to perform standardized comparisons or pooled interpretation [51]. The search was limited to a single database (PubMed), which may have omitted relevant studies indexed elsewhere. These limitations underscore that the apparent benefits of NAC, such as the potential role in supporting treatment de-escalation, should be considered hypothesis-generating rather than definitive (Figure 1).

Finally, in the context of therapeutic de-escalation, RCTs are needed to assess the role of NAC, as well as its benefits and drawbacks in patients with locally advanced HPV-positive or HPV-negative OPSCC that are surgically resectable or not. Future RCTs should incorporate stratification by HPV status; clear definitions of resectability; clear definition of oncologic endpoints; functional outcomes using standardized instruments; and long follow-up to capture late toxicity and recurrences. Based on current evidence, two key approaches warrant investigation: first, comparing upfront surgery followed by RT/CRT with NAC + S (and tailored adjuvant treatment); second, comparing CRT and salvage surgery with NAC + S (and tailored adjuvant treatment). For unresectable advanced tumors, another area of interest is comparing CRT with salvage surgery versus NAC followed by surgery in patients achieving tumor downstaging.

Until such data are available, generalizability of these findings to OPSCC populations remains uncertain and management should adhere to established treatment protocols within the framework of clinical trials.

## Figures and Tables

**Table 1:** Characteristics and response of early studies on NAC in OPSCC before Domenge et al.’s study [35].

Author	Number of Patients	T3-4 (%)	Chemotherapy Schema	Clinical Partial Response (%)	Clinical Complete Response (%)
Deitmer et al. * [30]	27	70	Cisplatin, Bleomycin, Methotrexate	77	22
**Calais et al. (Study 1)** [31]	135	NR	Cisplatin-based	39	6
**Calais et al. (Study 2)** [32]	138	NR	Cisplatin-based	34	NR
**Rohrmeier et al.** [33]	41	95	Cisplatin, Bleomycin, Methotrexate	12	10
**Bettinger et al.** [34]	43	NR	Bleomycin, Cisplatin	81	0
Pfister et al. * [6]	33	87	Cisplatin-based	64	42
Nathu et al. ** [7]	26	100	Cisplatin, 5-FU	62/69	35/50

* Primary tumor and lymph node combined response. ** Primary tumor/lymph node exclusive response.

**Table 2:** Characteristics and response of studies of NAC in OPSCC published after the Pignon meta-analysis but before the HPV association.

Author	Number of Patients	T3-4 (%)	Chemotherapy Schema	Partial Response (%)	Complete Response (%)
**Mantz et al.** [8]	61	NR	Platinum-based chemotherapy + concurrent CRT	NR	65
**Machtay et al.** [9]	53	78	Cisplatin, 5-FU + concurrent CRT	89	13
**Finnegan et al.** [37]	23	100	Cisplatin, 5-FU + hyperfractionated RT	74	NR
**Prestwich et al.** [38]	41	61	Cisplatin, 5-FU + sequential/concomitant CRT	68	10
**Urba et al.** [39]	37	75	Cisplatin, 5-FU + concurrent CRT	81	NR

**Table 3:** Characteristics and response of studies on NAC + surgery in OPSCC.

Author	Sadeghi et al. [21] (2016)	Sadeghi et al. [23] (2020)	Park et al. (2017) [22] Solimeno (2021) [40]	Costantino et al. (2023) [41]	Kim et al. (2024) [42]
**Number of Patients**	17	54	31	198	38
**Neoadjuvant Chemotherapy Schema**	Cisplatin, Docetaxel	Cisplatin, Docetaxel	Cisplatin + TS-1 (gimeracil + tegafur)	Cisplatin + TS-1	Docetaxel, Cisplatin, 5-FU
**p16+ (%)**	59	100	68	69	100
**T3-4 (%)**	47	31	100	34	18
**N2-3 (%)**	65	75	NR	78	26
**Primary tumor PR (%)**	NR	28	90	NR	100
**Nodal disease PR (%)**	NR	43	NR	NR	100
**Primary tumor CR (%)**	54	72	9.7	33	60
**Nodal disease CR (%)**	57	57	NR	43	45
**DFS (%)**	94.1 (3-y)	NR	80.8 (5-y)	81.4 (3-y)	64.2 (5-y)
**OS (%)**	94.1 (3-y)	NR	78.7 (5-y)	88.7 (3-y)	81.1 (5-y)
**Systemic recurrence** **(%)**	NR	NR	3.2	4.5	10.5
**Severe toxicity after NAC (%)**	17	NR	NR	NR	NR
**Adjuvant postoperative RT/CRT (%)**	31		87	60	58

**Table 4:** Characteristics and response of studies on NAC+CRT in OPSCC.

Author	Marur et al. (2016) [44]	Misiukiewicz et al. (2019) [45]	Seiwert et al. (2019) [46]	Rosenberg et al. (2021) [47]	Rosenberg et al. (2024) [26]
**Number of Patients**	80	20	62	90	73
**Neoadjuvant chemotherapy schema**	Cisplatin, Paclitaxel, Cetuximab	Docetaxel, Cisplatin, Fluorouracil	Carboplatin, Nab-Paclitaxel	Carboplatin, Nab-Paclitaxel/Paclitaxel	Nivolumab, Nab-Paclitaxel, Carboplatin
**p16+ (%)**	96	80	100	100	100
**T3-4 (%)**	25	45	32	34	40
**N2-3 (%)**	85	65	82	95	91
**Primary tumor PR (%)**	9 *	20	27	88	97
**Nodal disease PR (%)**	58 *	15	NR	NR	NR
**Primary tumor CR (%)**	70 *	80	71	NR	70.8
**Nodal disease CR (%)**	58 *	80	90 **	NR	NR
**DFS (%)**	78 (2-y)	87.5 (3-y)	94.5 8 (2-y)	90 (5-y)	90 (2-y)
**OS (%)**	91 (2-y)	83.5 (3-y)	98 (2-y)	90 (5-y)	91.4 (2-y)
**Systemic recurrence (%)**	1	0	5	1	1
**Severe toxicity after NAC (%)**	23	25	37	40	5
**RT de-escalation**	77	60	80	83	86

* clinical response; ** pathological response.

## Data Availability

The data presented are available in the references cited in this article.
